# Friday Night Acute Kidney Injury: Chest Infection Masking Antineutrophil Cytoplasmic Antibody Vasculitis

**DOI:** 10.7759/cureus.90094

**Published:** 2025-08-14

**Authors:** Rawaha Ahmad, Noor Un Nahar, Ismaa Kiani, Muqaddas Imran, Muhammad Umair Sultan

**Affiliations:** 1 Acute Medicine, Northampton General Hospital, Northampton, GBR; 2 Nephrology, Northampton General Hospital, Northampton, GBR; 3 General Medicine, Northampton General Hospital, Northampton, GBR; 4 Internal Medicine, Northampton General Hospital, Northampton, GBR

**Keywords:** #aki, anca-associated vasculitis, granulomatosis with polyangiitis (gpa), renal pathology, small vessel vasculitis

## Abstract

Antineutrophil cytoplasmic antibody (ANCA)-associated vasculitis (AAV) includes a group of autoimmune disorders resulting in the production of autoantibodies to neutrophil proteins (leukocyte proteins, proteinase 3 (PR3)-ANCA or myeloperoxidase proteins (MPO)-ANCA). There are three subtypes of AAV based on ANCA serotypes: PR3+ AAV, MPO+ AAV, and ANCA+. These rare disorders present with a wide spectrum of symptoms with involvement of multiple organs, including the heart, kidneys, eyes, ears, brain, spinal cord, nerves, and musculoskeletal system, making them difficult to diagnose promptly. This impacts the prognosis of AAV, as involvement of major organs can increase recurrence rates and lead to a poor prognosis, while timely diagnosis can lead to full recovery. Treatment options mainly involve immunosuppression with steroids, rituximab, methotrexate, and cyclophosphamide to maintain remission. We discuss a case of granulomatosis with polyangiitis in an adult male. We believe this report will help highlight timely diagnosis and interventions, overall improving the prognosis of patients.

## Introduction

Antineutrophil cytoplasmic antibody (ANCA)-associated vasculitis (AAV) comprises a rare group of disorders with a worldwide incidence of 18.4 per 100,000 in Germany, Australia, Sweden, and the USA: an increase from 4.6 per 100,000 over the past 30 years [1]. This increase is either a true increase in prevalence or improved diagnostic capabilities. Data from the UK report a granulomatosis with polyangiitis (GPA) incidence of 9.97 per 100,000 [2].

ANCA-associated vasculitis (AAV) affects small- to medium-sized vessels throughout the body in a relapsing pattern, often involving multiple organs. This results in a wide array of symptoms [3]. Given the nonspecific nature of these symptoms, which can mimic common clinical presentations, we emphasize the importance of maintaining a broad differential when assessing patients. The kidneys and lungs are the most commonly affected organs and often determine the overall prognosis [4]. Due to the underlying pathophysiology, treatment primarily focuses on immunosuppression. Early initiation of therapy is crucial to prevent long-term complications and induce remission [3].

## Case presentation

This patient is a 73-year-old English male who presented to the emergency department with shortness of breath and was diagnosed with bilateral community-acquired pneumonia and acute kidney injury (AKI) secondary to sepsis. He was referred to the medical team, where I saw the patient.

Upon taking the history, the patient reported shortness of breath and a productive cough mixed with blood for the past five days. He had consistent symptoms with no exacerbating or relieving factors. He denied having fever, malaise, sore throat, flu-like illness, headache, night sweats, changes in appetite or weight, chest pain, joint pain, rash, redness and watery eyes, or any urinary or bowel changes. There was no previous similar episode in himself or a similar illness in the family. He denied any toxic substance or recreational drug abuse. He had a past medical history of ocular hypertension, for which he was on follow-up with ophthalmology. He had no regular medications prescribed.

On examination, the patient was an adult man lying in bed, receiving 5 L of oxygen via nasal cannula. His blood pressure was 100/60 mmHg, respiratory rate was 26 to 28 breaths per minute, and heart rate ranged from 110 to 150 beats per minute. Physical examination was unremarkable except for bilateral crackles heard over the posterior chest.

Table 1 shows the first blood tests received by the laboratory, indicating that the patient had anemia and a marked inflammatory response, with raised white cell count (WCC), neutrophils, and CRP, suggesting possible infection or systemic inflammation. Renal function was impaired, indicated by elevated creatinine and low estimated GFR (eGFR). The very high D-dimer raised further concerns of severe inflammatory processes.

**Table 1:** Blood results on arrival in A&E. A&E: accident and emergency

Test	Result	Reference Range
Hemoglobin (Hb)	7.7 mmol/L	8.6–11.2 mmol/L (↓ Low)
White Cell Count (WCC)	12.9 x 10⁹/L	4.0–11.0 × 10⁹/L (↑ Elevated)
Neutrophils	11.12 x 10⁹/L	2.0–7.5 × 10⁹/L (↑ Elevated)
Eosinophils	0.01 x 10⁹/L	0.0–0.5 × 10⁹/L (Within normal limits)
Lymphocytes	1.02 x 10⁹/L	1.0–3.5 × 10⁹/L (Slightly low/normal)
Monocytes	0.69 x 10⁹/L	0.2–0.8 × 10⁹/L (Within normal limits)
C-Reactive Protein (CRP)	152 mg/L	< 0.3 mg/dL (↑ Markedly elevated)
Estimated GFR (eGFR)	38 mL/min/1.73 m²	≥ 90 mL/min/1.73 m² (↓ Decreased)
Creatinine	153 µmol/L	62–115 µmol/L (↑ Elevated)
D-Dimer	5,884 ng/mL	<500 ng/mL(↑↑ Significantly elevated)

**Figure 1:**
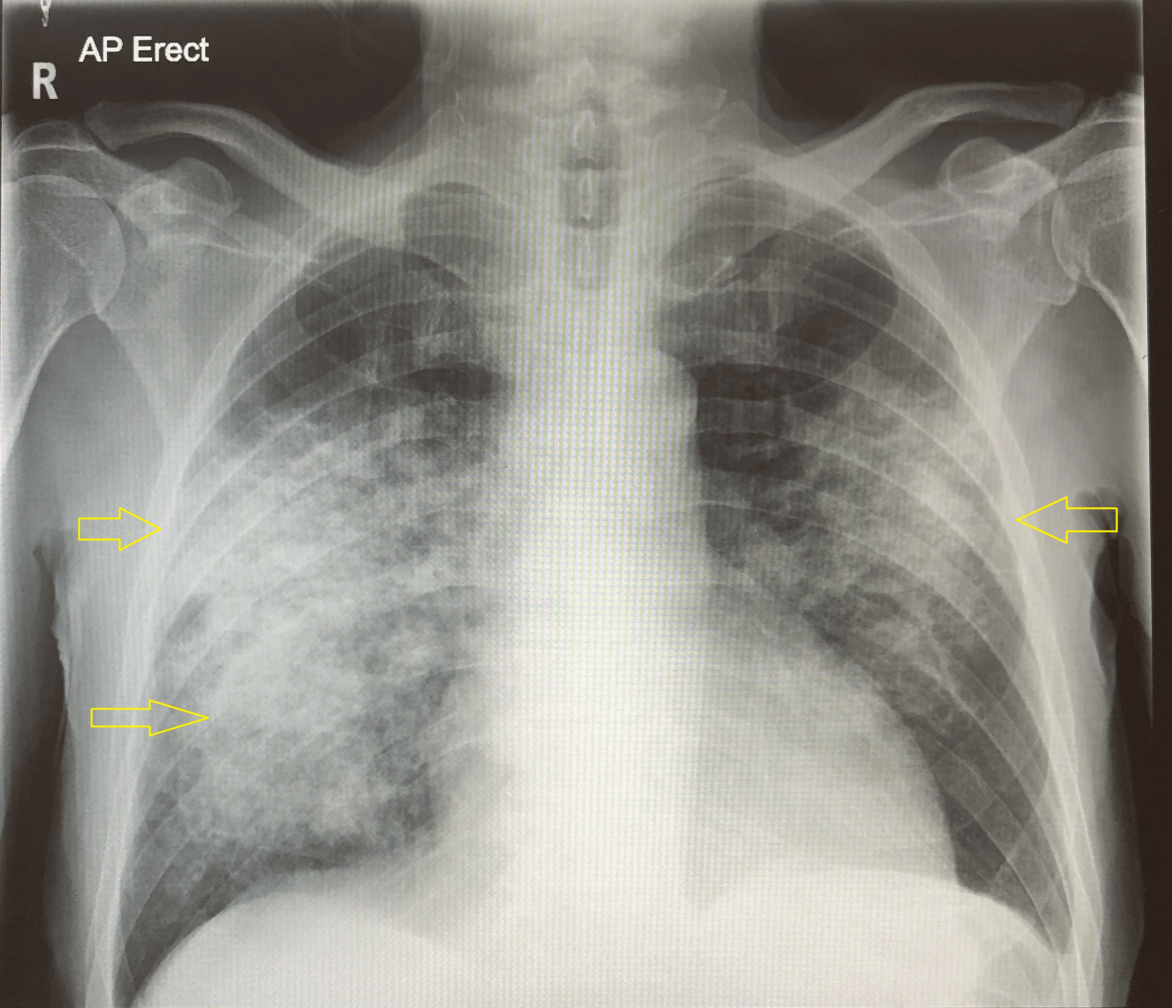
Chest X-ray showing bilateral widespread multifocal airspace shadowing, more prominent on the right. Trivial blunting of the left costophrenic angle.

Figure 1 is an AP view imaging of the chest, showing bilateral airspace shadowing (white) that is more prominent in the right lung. The configuration may suggest pulmonary edema, hemorrhage, or inflammatory changes. In the left lung, there is a slight blunting of the costodiaphragmatic angle.

After summarizing notes of the patient, we requested a basic bedside urine dipstick test, which showed blood (+++) and protein (++). This urine picture urged us to send an autoimmune screen of the patient while providing supportive treatment. Table 2 contains the results from the urine analysis and autoimmune screen.

**Table 2:** Autoimmune profile and urine analysis results. GBM: glioblastoma; ANA: antinuclear antibody; PR3: proteinase 3; MPO: myeloperoxidase; HPF: high-pass filter

Test	Result	Interpretation
Autoimmune Profile		
Anti-GBM	< 2.9 U/mL	Negative (normal < cutoff)
ANA	Negative	Negative
Anti-PR3	> 3,285 U/mL	Elevated (significant increase)
Anti-MPO	< 3.2 U/mL	Negative (normal < cutoff)
Infection Marker		
Procalcitonin	0.47 ng/mL	Normal to mildly elevated (cutoff usually <0.5)
Urine Dipstick		
Blood	+++	Positive (significant hematuria)
Protein	++	Positive (proteinuria)
Urine Analysis		
White Cells (WBC)	164 cells/HPF	Elevated (normal usually < 10-15 cells/HPF)
Red Blood Cells (RBC)	69 cells/HPF	Elevated (normal usually < 3-5 cells/HPF)
Epithelial Cells	< 10 cells/HPF	Within normal limits
Urine Protein Creatinine Ratio	49 mg/mmol	Elevated (proteinuria)
Blood Cultures	Negative	No bacterial growth detected

**Figure 2:**
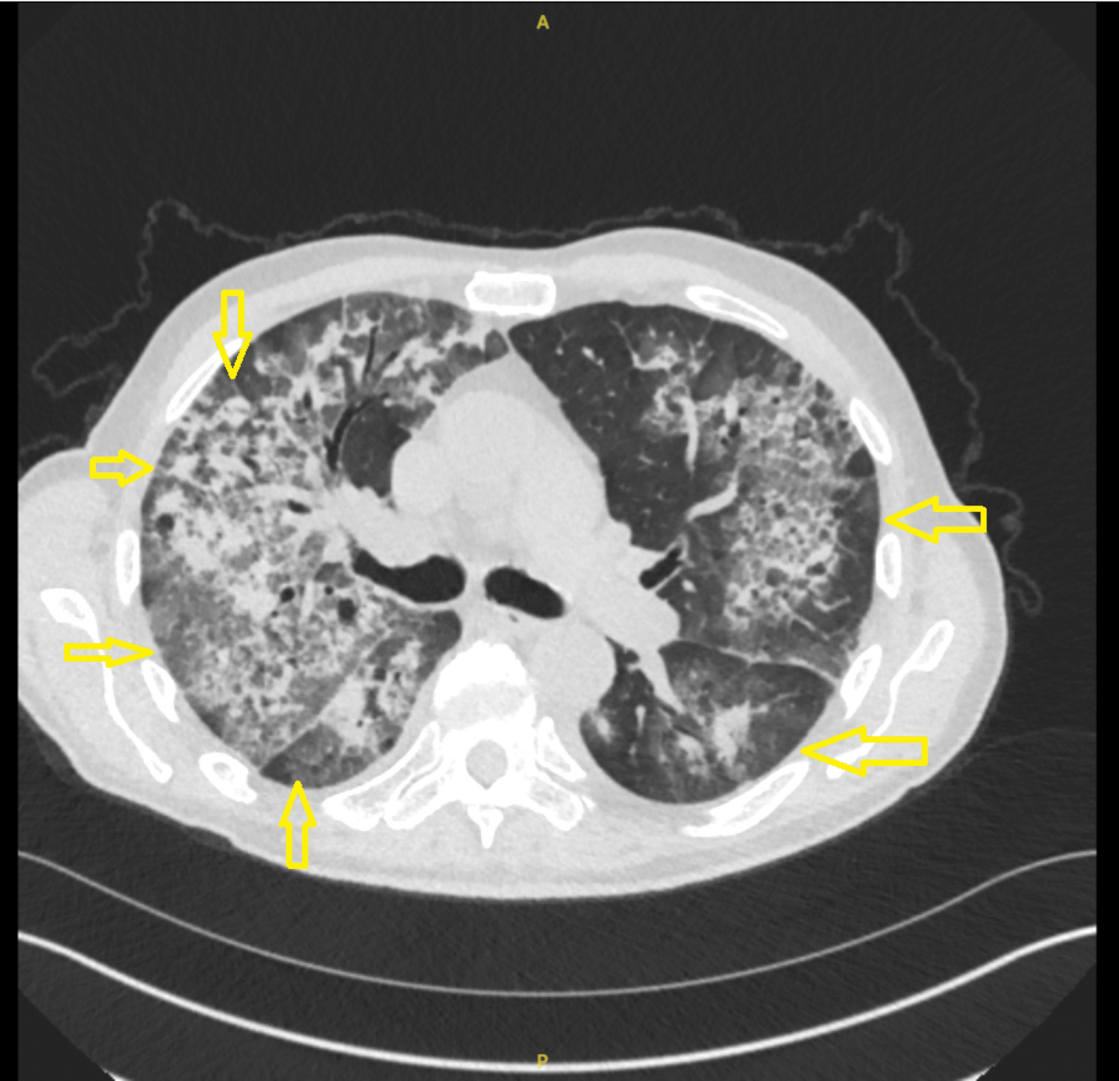
CT chest showing bilateral widespread pulmonary consolidations, more pronounced on the right side; findings suggest the need to rule out pulmonary edema, hemorrhage, viral pneumonia, or Pneumocystis pneumonia.

Figure 2 is a CT scan urgently performed, reported as showing bilateral widespread pulmonary consolidations, more pronounced on the right side, with a central batwing appearance and some peripheral ground-glass changes. There is a background of centrilobular pulmonary emphysema. Mild bilateral pleural effusion is also noted.

His ANCA profile returned highly positive for proteinase 3-positive (PR3+) ANCA. Considering his hemodynamic compromise, a renal biopsy was not done, and he was transferred to the ICU and started on intravenous methylprednisolone (1 g stat) and prednisolone (60 mg per oral once daily), along with seven sessions of plasmapheresis. This resulted in marked clinical improvement. He was prescribed a three-month maintenance course of cyclophosphamide (100 mg orally once daily) and a tapering dose of prednisolone to a 5 mg maintenance dose once daily.

Table 3 comprises the trend of creatinine, adding the values of alternate days.

**Table 3:** Creatinine trend on alternate days.

Day	Creatinine (µmol/L)
Day 1	153 µmol/L
Day 3	180 µmol/L
Day 5	237 µmol/L
Day 7	217 µmol/L
Day 9	166 µmol/L
Day 11	130 µmol/L
Day 13	120 µmol/L

**Figure 3:**
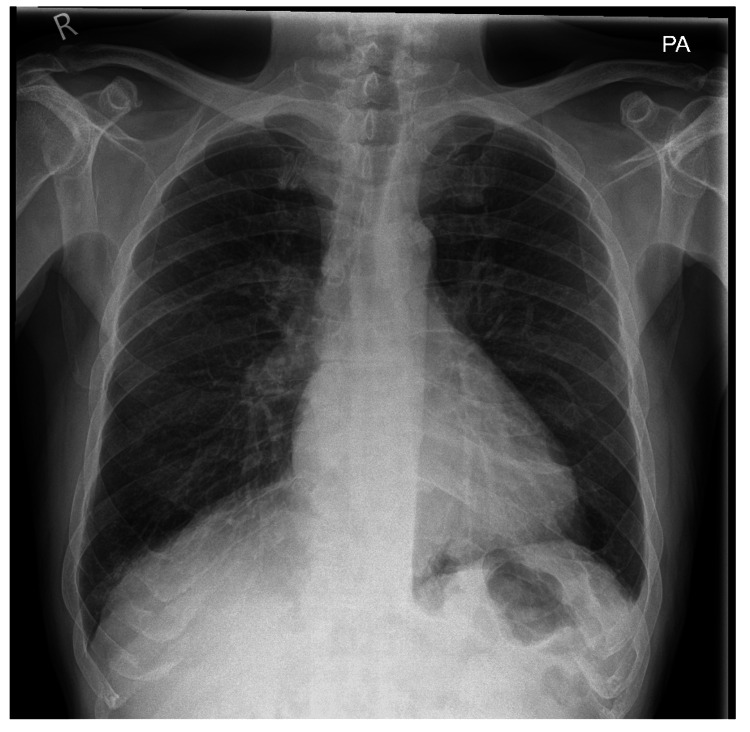
Follow-up outpatient department chest X-ray done at four months after discharge.

Within two weeks, his AKI had resolved, and he was discharged with a follow-up appointment in the day case clinic. His labs had significantly improved, and he was referred for a follow-up at the vasculitis clinic. Figure 3 shows the follow-up chest X-ray done in the rheumatology clinic at four months, which, in comparison, showed significant improvement and resolution of shadowing bilaterally.

## Discussion

Shortness of breath and cough are common presentations of lower respiratory tract infections or pneumonia, but they are not specific to these conditions. Therefore, thorough history-taking and investigations guided us toward the possibility of an autoimmune disorder.

GPA is one of the subtypes of ANCA-associated vasculitis disorders that include PR3+, myeloperoxidase-positive (MPO+), and ANCA +/- eosinophilic variants [1]. AAV occurs when primed vascular neutrophils are activated by the binding of ANCA (predominantly immunoglobulin G (IgG)) present in plasma to ANCA autoantigens in neutrophils via Fcγ and F(ab’)2 receptors, triggered by factors such as genetic predisposition (e.g., HLA-DRB1*15 allele for GPA), loss of B- and T-cell tolerance, environmental factors, response to injury, and drug-induced mechanisms [3]. An alternative complement pathway is activated by these neutrophils, producing C5a, which further attracts additional neutrophils. These neutrophils eventually adhere to vessel walls, releasing toxins and free radicals that cause necrosis of neutrophils and damage to the endothelial lining of vessels [4].

Multiple acute lesions are present in AAV patients, who will transition from the acute to the chronic phase within one to two weeks. Similarly, monocytes with ANCA autoantigens are activated, producing monocyte chemoattractant protein 1/chemokine ligand 2 (MCP-1/CCL2) and interleukin-8 (IL-8), further transforming the process into granulomatous inflammation [5,6].

Another theory for AAV pathology is an autoimmune response to complementary autoantigens. As in PR3+ ANCA vasculitis, formation of autoantibodies occurs against PR3+ due to autoantibodies targeting complementary PR3+ (c-PR3+) peptides. Both are generated by the sense and antisense strands of the PR3 gene. Therefore, antibodies against c-PR3 will act against the original PR3 [7].

Patients with GPA can present with a wide array of symptoms, ranging from upper to lower respiratory tract involvement, renal involvement, recurrent otitis media, sinusitis, gastrointestinal (GI) ulcerations, mononeuritis multiplex, cutaneous presentations, and even pericarditis or conduction abnormalities. Ophthalmic involvement, such as recurrent scleritis, keratitis, renal vessel occlusion, and nasolacrimal stenosis, is present in half of the patients. In contrast, our patient had an acute presentation with a five-day history and no significant prior history [8].

Diagnosis of GPA is made based on serum ANCA, biopsy, and imaging of involved organs [9]. The mainstay of treatment is immunosuppressive therapy with steroids and cytotoxic drugs. The combination of steroids and cyclophosphamide is the treatment of choice for the induction of remission [10].

Alternatively, combinations of steroids with rituximab (anti-CD20 antibody) are more effective in terms of fewer side effects. Patients who undergo plasma exchange and ANCA removal show a better prognosis in terms of kidney function. Ultimately, the goal is to induce remission and prevent relapse, which requires ongoing monitoring [11].

## Conclusions

Although AAV presentations are nonspecific, timely diagnosis and immediate intervention are key aspects of managing vasculitis. This paper emphasizes the importance of remaining mindful of possible autoimmune disorders in patients presenting with such symptoms and performing bedside tests, such as urinalysis, which can help diagnose patients within 24 hours and initiate the best possible management.

This report aims to enhance knowledge among medical practitioners regarding AAV and to emphasize the importance of timely diagnosis in greatly improving patient outcomes. From requiring 5 L of oxygen to being discharged within two weeks, we cannot emphasize enough the importance of the right diagnosis at the right time, followed by the right intervention.
